# Does preliminary optimisation of an anatomically correct skull-brain model using simple simulants produce clinically realistic ballistic injury fracture patterns?

**DOI:** 10.1007/s00414-017-1557-y

**Published:** 2017-03-07

**Authors:** P. F. Mahoney, D. J. Carr, R. J. Delaney, N. Hunt, S. Harrison, J. Breeze, I. Gibb

**Affiliations:** 1 Royal Centre for Defence Medicine, ICT Centre, Research Park, Birmingham, B15 2SQ UK; 2 Centre for Defence Engineering, Cranfield University at the Defence Academy of the United Kingdom, Shrivenham, Swindon, SN6 8LA UK; 3 South West Forensic Pathology Group Practice, PO Box 388, Bristol, BS9 0DB UK; 4 Forensic Pathology Services, Grove Technology Park, Wantage, Oxon OX12 9FA UK; 5 Academic Department of Military Surgery and Trauma, RCDM, Birmingham, UK; 6 X-ray Department, Medical Centre, HMS Nelson, HM Naval Base Portsmouth, Hampshire, PO1 3HH UK

**Keywords:** Head, Military helmet, Assessment, 7.62 × 39 mm bullet, AK47

## Abstract

Ballistic head injury remains a significant threat to military personnel. Studying such injuries requires a model that can be used with a military helmet. This paper describes further work on a skull-brain model using skulls made from three different polyurethane plastics and a series of skull ‘fills’ to simulate brain (3, 5, 7 and 10% gelatine by mass and PermaGel™). The models were subjected to ballistic impact from 7.62 × 39 mm mild steel core bullets. The first part of the work compares the different polyurethanes (mean bullet muzzle velocity of 708 m/s), and the second part compares the different fills (mean bullet muzzle velocity of 680 m/s). The impact events were filmed using high speed cameras. The resulting fracture patterns in the skulls were reviewed and scored by five clinicians experienced in assessing penetrating head injury. In over half of the models, one or more assessors felt aspects of the fracture pattern were close to real injury. Limitations of the model include the skull being manufactured in two parts and the lack of a realistic skin layer. Further work is ongoing to address these.

## Introduction

Ballistic head injury remains a significant threat in modern conflict. Smith et al. [1] undertook a retrospective database review of patients presenting to UK field hospitals in Iraq and Afghanistan between 2003 and 2011. Eight hundred and thirteen patients on the database had suffered a penetrating head injury. Gunshot wound (GSW) was associated with a more severe injury and worse outcome than blast fragment injury. One of the study conclusions was that further work is needed to understand both the underlying anatomical lesions and the energy transfer distribution.

A further study [2] undertook a retrospective review of 71 casualties in Iraq and Afghanistan who had reached medical treatment facilities alive but subsequently died of their wounds. The most common cause of death (44 out of 71) was severe head injury from explosion, blast fragmentation and GSWs. Analysis of 42 of the patients (where full records were available) found that improved medical care would not have helped them and work should be concentrated on improving head protection.

All UK deaths on deployed operations are reviewed by a multidisciplinary panel [3]. A key output from these reviews has been identifying new injury patterns and informing the ongoing development of personal protective equipment.

Understanding and investigating these injury mechanisms and potentially suggesting improvements in head protection require suitable models.

There are many physical models used to assess head injury described in the literature. These include post mortem human specimens [4], anaesthetised animals [5], animal material [6] and synthetic materials [7].

Thali et al. [7] described development of a ‘skin-skull-brain model’ consisting of a ‘scalp’ made from silicon, a layered polyurethane sphere to represent the skull and gelatine 10% at 4 °C to simulate brain. The model was shot with a series of ammunition types and the authors reported that ‘injuries inflicted to this model are fully comparable to the morphology of equivalent real gunshot injuries’.

Raymond and Bir [8] assessed a similar model against post mortem human specimens using blunt impacts (a 103-g rigid impactor at 20 m/s) but found the fracture patterns to be different in the human bone compared to the polyurethane spheres.

Bir et al. [9] assessed two different synthetic femurs (SAWBONES® and SYNBONE®) against post mortem human material looking at both direct and indirect fractures from ballistic events and used a trained trauma surgeon to assess the injuries. The SYNBONE® produced similar fracture patterns to the human material but needed a higher direct impact velocity to create this. The SAWBONES® fracture patterns were different to the human material. The authors concluded that the bone surrogates did not approximate to the cadaveric bone under the experimental conditions used.

There are ethical and practical issues around the use of cadavers and animals which makes synthetic bone substitutes an attractive and practical option [10]. Smith et al. [10] subjected SYNBONE® polyurethane bone substitute (flat plates and spheres) to a series of ballistic impacts (0.243 in. Winchester Soft Point, 7.62 × 51 mm NATO Full Metal Jacket, 13.5 mm solid lead ball and 8.0 mm Perfectline alloy cross bow bolt) and compared both the macroscopic appearances and the microscopic damage to experimental animal bone samples and published examples of human injury. They noted clear differences between real bone and the synthetic materials but felt the SYNBONE® spheres offered a useful approximation to the damage seen in bone.

Thali et al. [7] stated that they chose a SYNBONE® sphere rather than a more complex skull form as it would offer ‘more reproducible and comparable results’. A sphere is, however, not suitable for studies incorporating helmets.

Preliminary work to develop a suitable anatomically correct skull brain model for ballistic studies incorporating a helmet have been reported [11]. This model consisted of an anatomically correct polyurethane skull with a 10% gelatine brain (by mass; 4 °C) impacted with 7.62 × 39 ammunition (M43 ball, Chinese, mild steel core, Factory 71, 1984) at a mean velocity of 675 m/s. Only six results were reported, but the fracture patterns generated were compared to the limited forensic anthropology literature that exists and demonstrated macroscopic similarities [12, 13].

The aim of this subsequent work was to assess if the fracture patterns produced under a series of further experimental conditions using simple simulants would be assessed as realistic by clinical experts.

## Method

The research described in this paper was carried out in a number of stages.

Anatomically correct polymeric skulls were manufactured from rapid prototype data obtained by 3D mapping of both the internal and external surfaces of a human skull (ARRK Europe Ltd., Gloucester Technical Centre, Olympus Park, Quedgeley, Gloucester, Gloucestershire GL2 4NF).

- The first stage (*n* = 9 skulls) involved a comparison of skulls made from different polymers (Table 1).

**Table 1:** Summary of synthetic skull data

Polymer	Hardness shore (D)	Tensile strength (MPa)	Bending strength (MPa)	Impact strength (kJ/m^2^)	Number
PU8098	85	70	75	10	3
UP5690	83	35	50	100	3
MU51	81	54	87	13	3

Data from material manufacturers provided to ARRK Europe Ltd. Craig Vickers, Personal communication, January 2017

The two parts of the skull were bonded using cyanoacrylate adhesive (Loctite, Henkel Corp., USA). A thin low-density polyethylene bag was inserted into the base of the skull and gelatine, 10% by mass, poured into the bag to fill the cranial cavity. The gelatine was allowed to set for 24 h at 17 °C (laboratory temperature) and then conditioned at 4 °C for a further 24 h [11].

b. The second stage (*n* = 30 skulls) involved a comparison of different fills and conditioning temperatures. With regard to simulating brain tissue, two of the authors (PM and SH) felt that gelatine 10% seemed too stiff when compared to living brain tissue in recently ballistically injured casualties. This was the incentive to explore the behaviour of different gelatine concentrations. Gelatine 10% by mass has been used extensively in ballistic experiments, but there is still uncertainty as to how it relates to biological tissue [14].

Skulls made of polymer MU51 were filled with either gelatine made by mass to 3, 5, 7 and 10% or with PermaGel™.

The skulls were filled as described in the “Methods (item a)” section (above) and again allowed to set overnight at 17 °C. The gelatine was then either:

i. conditioned for a further 24 h at 4 °C and removed from the fridge just before being shot or
ii. allowed to remain at 17 °C until shot or
iii. kept in an oven at 25 °C until shot.

Permagel™ is reportedly a remeltable and reusable ballistic test material equivalent to 10% gelatine, although ballistic testing with steel spheres has suggested that PermaGel™ is strain rate sensitive and its properties vary between 10 and 20% gelatine [15]. Permagel™ is melted at 110 °C, and therefore, a thin oven ‘roasting bag’ was used to contain the molten Permagel™ rather than the polyethylene bag used for the liquid gelatine. Once poured into the skulls, the Permagel™ was allowed to cool to 17 °C (laboratory temperature) and remained at this temperature until shot.

The different fill and temperature combinations are summarised in Table 2.

**Table 2:** Summary of skull ‘fill’ (gelatine % by mass or Permagel™) and temperature of fill immediately after ballistic impact

Skull ‘fill’	Temperature (rounded) (°C)	Number
Gelatine 10%	4	5
Gelatine 10%	17	3
Gelatine 7%	17	5
Gelatine 5%	17	3
Gelatine 3%	4	2
Gelatine 3%	17	3
Gelatine 3%	25	3
Permagel™	17	6

c. The first nine skulls were shot with 7.62 × 39 mm Czech mild steel core ammunition (Sellier and Bellot, Prague; Factory in Zbrojovka Vlàsim, manufactured 1983, mean muzzle velocity 708 m/s, SD = 9 ms). The next 30 were shot with 7.62 × 39 mm Ukrainian mild steel core ammunition (Soviet State Factory, Lugansk, manufactured 1967, mean muzzle velocity 680 m/s, SD = 24 m/s) (Fig. 1).

**Fig. 1:**
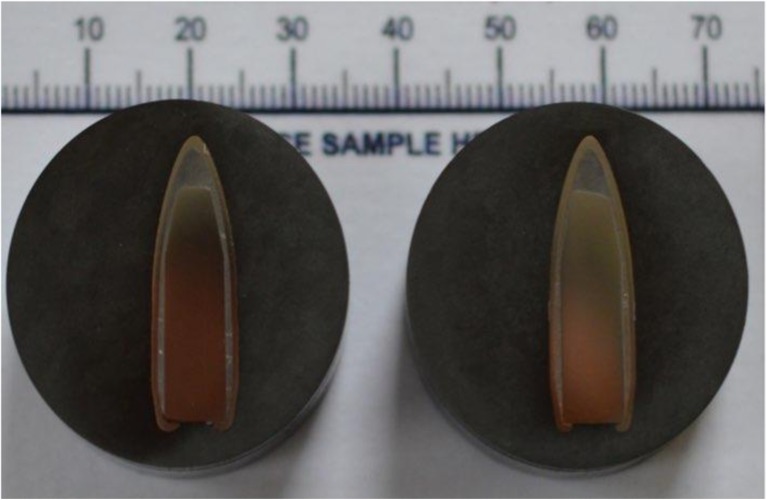
Sectioned 7.62 × 39 mm bullets. *Left*, Czech; *right*, Ukrainian

**Fig. 2:**
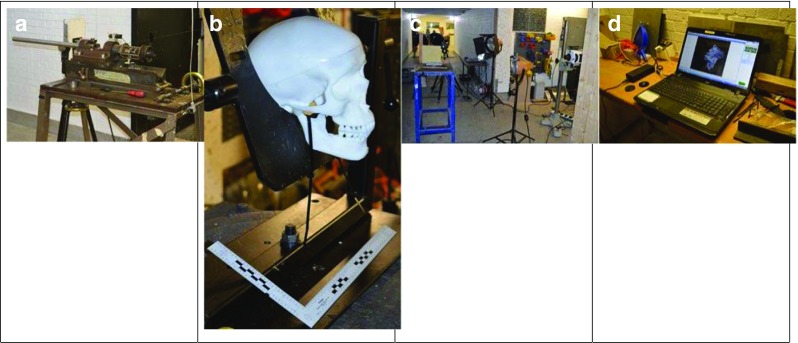
Experimental setup. **a** Enfield proof mount. **b** Skull-brain model. **c** Camera and lighting setup. **d** Image capture on laptop PC

The models were shot at a range of 10 m from a no. 3 Enfield proof mount fitted with an accurate barrel (Fig. 2a). Prior to each shot, the impact site on the model (Fig. 2b) was confirmed using a sighting laser. Bullet velocity was tracked using a Weibel Doppler and impacts filmed using two Phantom high speed cameras (V12 and V1212) (Fig. 2c, d).

The condition of the models in situ post impact was captured using a Nikon D3200 DSLR camera fitted with an AF-S NIKKOR 18–55-mm lens. The fractured skull pieces and gelatine or Permagel™ contents were collected post impact and the extent of the damage recorded. The temperature of the gelatine and Permagel™ was recorded immediately post impact using a calibrated digital thermometer (Table 2).

d. The third stage was inviting five military and civilian clinicians with extensive experience of managing and/or assessing ballistic head injury to individually assess the fracture patterns in the skulls and score how clinically realistic they were using a four-point Likert-type scale [16] (Table 3).

**Table 3:** Likert-type scale for expert assessments

1. This looks nothing like a real fracture pattern
2. This looks a bit like a real fracture pattern
3. This looks a lot like a real fracture pattern
4. This looks exactly like a real fracture pattern

**Fig. 3:**
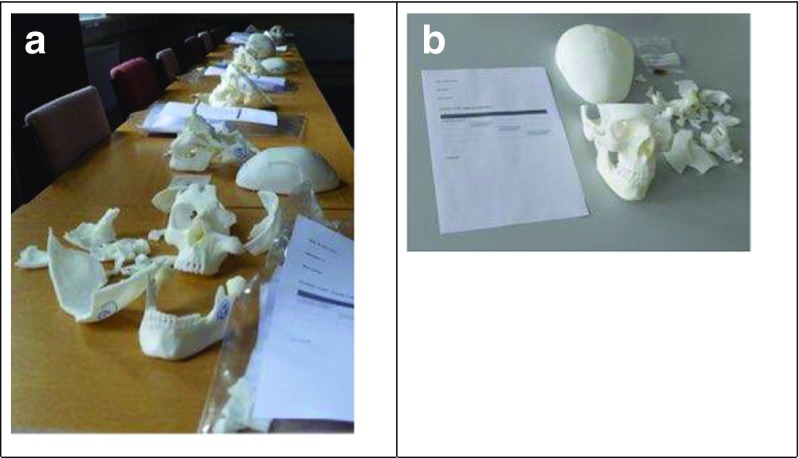
Shot skull assessment setup. **a** Skull assessment stations. **b** Individual skull with score sheet

The score sheet also included space for comments if the clinician wished to provide them (Fig. 3a, b).

The clinicians invited to assess the skull models have either looked after casualties with ballistic head injury, reviewed x-ray and CT images from such casualties or conducted post mortem examinations of fatalities. Two had been regular members of the Mortality Peer Review Panel, a multidisciplinary group undertaking peer review of UK military deaths including the nature of the injuries and treatment given [3]. The current study was an opportunity to harvest this extensive collective knowledge.

The backgrounds of the clinicians were two Civilian Home Office Forensic Pathologists, and a military radiologist, neurosurgeon and maxillofacial surgeon. The clinicians were briefed on the bullet type used (i.e. 7.62 × 39 mm) and that different polymers/gelatine concentrations had been shot but were not given the details of the gelatine concentrations with individual skulls. The Permagel™ brains do not degrade the way gelatine does and were presented at the assessments with the skulls. No formal training in an assessment method was given; the clinicians were invited to score the skulls based on their own prior experience.

## Results

**Fig. 4:**
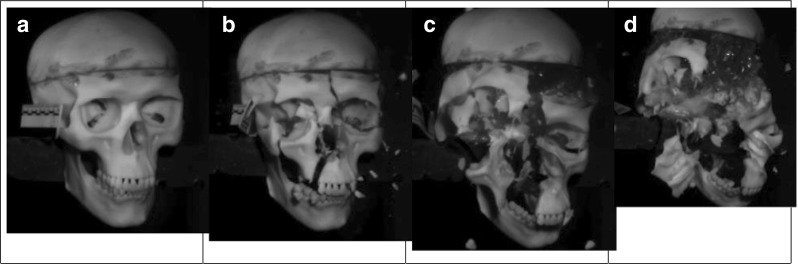
Frontal impact sequence. **a** 0 ms. **b** 12.25 ms. **c** 16 ms. **d** 23.93 ms

A typical fracture sequence captured with the V12 camera is shown in Fig. 4a–d.

The scores from the Likert-type scales were summarised in an Excel spreadsheet and analysed using International Business Machines Corporation’s Statistical Package for Social Services (IBM SPSS) version 23.

The free-text comments and notes made on the score sheets by the clinicians were also transcribed into an Excel spreadsheet so that comments about the wound characteristics and fracture patterns could be compared and assessed.

### IBM SPSS analysis

The effect of polymer type on fracture score was determined using analysis of variance (ANOVA); homogeneity and normality of data were checked, and a significance level of 0.05 was applied. Significant differences were identified using Tukey’s honest significant difference (HSD) test. Mean and standard deviation data are provided in Table 4.

**Table 4:** Descriptive statistics for the effect of polymer type on Likert-type score for fracture pattern

Polymer type	Mean Likert-type score for fracture pattern	Standard deviation
MU51	1.87	0.83
PU8098	1.93	0.70
UP5690	2.01	0.70

*N* = 15 assessor observations for each polymer

As shown, there was minimal difference among the scores for the different polymer types and the ANOVA found that polymer type did not affect fracture score (*F*_2,42_ = 0.28, *p* = NS).

The effect of gelatine concentration (or use of PermaGel™) and temperature (rounded) on the fracture score was similarly assessed. For the purpose of analysis, temperatures between 17 and 19 °C were rounded to 17 °C. Mean and SD data are provided in Table 5.

**Table 5:** Descriptive statistics for the effect of skull contents and temperature on fracture score

Gelatine (%)	Temperature (°C)	Mean Likert-type score for fracture pattern	Standard deviation	Number (assessor observations)
10	17	2.47	0.83	15
10	4	2.12	0.78	25
3	17	1.87	0.52	15
3	25	2.0	0.53	15
3	4	2.2	0.92	10
5	17	2.13	0.52	15
7	17	2.4	0.71	25
PermaGel™	17	2.1	0.71	30

ANOVA found that gelatine concentration (or use of PermaGel™) and temperature did not affect fracture score; gelatine/PermaGel™ (*F*_4,142_ = 1.21, *p* = NS); temperature (*F*_2,142_ = 0.01, *p* = NS)

### Summary of Likert-type scores and free-text comments

Of the 39 skulls assessed, 23 were given a score of 3 by at least one assessor and seven a score of 4 by at least one assessor.

No skulls received the same scores from all five assessors.

Thirteen skulls had more than one score of 3 or 4 from separate assessors and are summarised in Appendix Table 7. Assessor comments, where given, are included to demonstrate the elements that they felt were or were not representative of real injury.

In addition to their overall Likert-type fracture pattern score, assessors also commented on how realistic some of the entry and exit wounds appeared, along with the impact of the post mortem cut line.

**Fig. 5:**
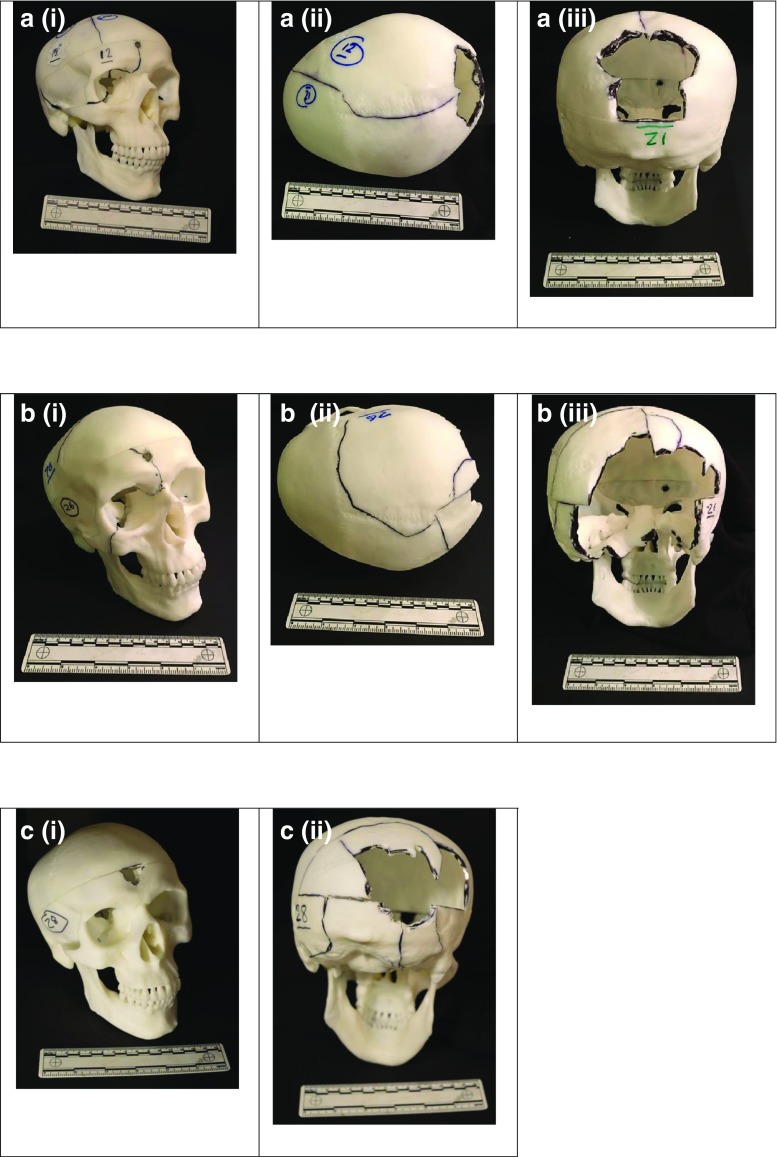
Examples of impacted skulls. The skulls have been reconstructed where possible. Fracture lines are highlighted using *black ink*. The skull numbers correspond to those in Appendix Table 7. **a** Skull 12, entry wound and associated fracture lines (*i*); skull 12, view from above. Fracture line and exit site (*ii*); skull 12, exit site, looking through to rear aspect of entry wound (*iii*). **b** Skull 26, entry site and associated fracture lines (*i*); skull 26, view from above (*ii*); skull 26, exit site, looking through towards rear aspect of entry wound (*iii*). **c** Skull 28, entry site (*i*); skull 28, exit site, looking through towards rear aspect of entry wound (*ii*)

The frequency of comments is summarised in Table 6. Examples of impacted skulls are shown in Fig. 5.

**Table 6:** Frequency of comments on entry and exit wound appearance plus influence of the PM cut line

Assessor	Entry realistic	Entry unrealistic	Exit realistic	Exit unrealistic	Number of occasions cut line interferes with fracture pattern
1	2	8	7	3	8
2	15	20	21	14	1
3	5	9	5	10	21
4	6	19	6	9	13
5	3	26	22	14	27

## Discussion

Smith et al. [10] found that spheres filled with ballistic gelatine produced damage patterns that compared well with published examples of real gunshot trauma, similar to the findings of both Thali et al. [7] using spheres and Carr et al. [11] using synthetic skulls. In this present work, the overall fracture patterns in 23 of the 39 skulls (59%) were considered close to reality by at least one of the five assessors.

There were differences of opinion. For example, in two skulls (numbers 8 and 11), non-pathologists commented that the fracture patterns were too extreme to reflect reality. These were both scored high by one pathologist and the radiologist. The published literature includes cases of similar devastating head injury [17]. The useful observation is that experts will interpret based on past experience which needs to be matched to the injury being investigated or modelled.

The majority view from the clinicians was that many of the entrance wounds were not realistic. The ‘classical’ appearances of gunshot wounds to the skull are described in a number of forensic science and pathology text books [18–20].

**Fig. 6:**
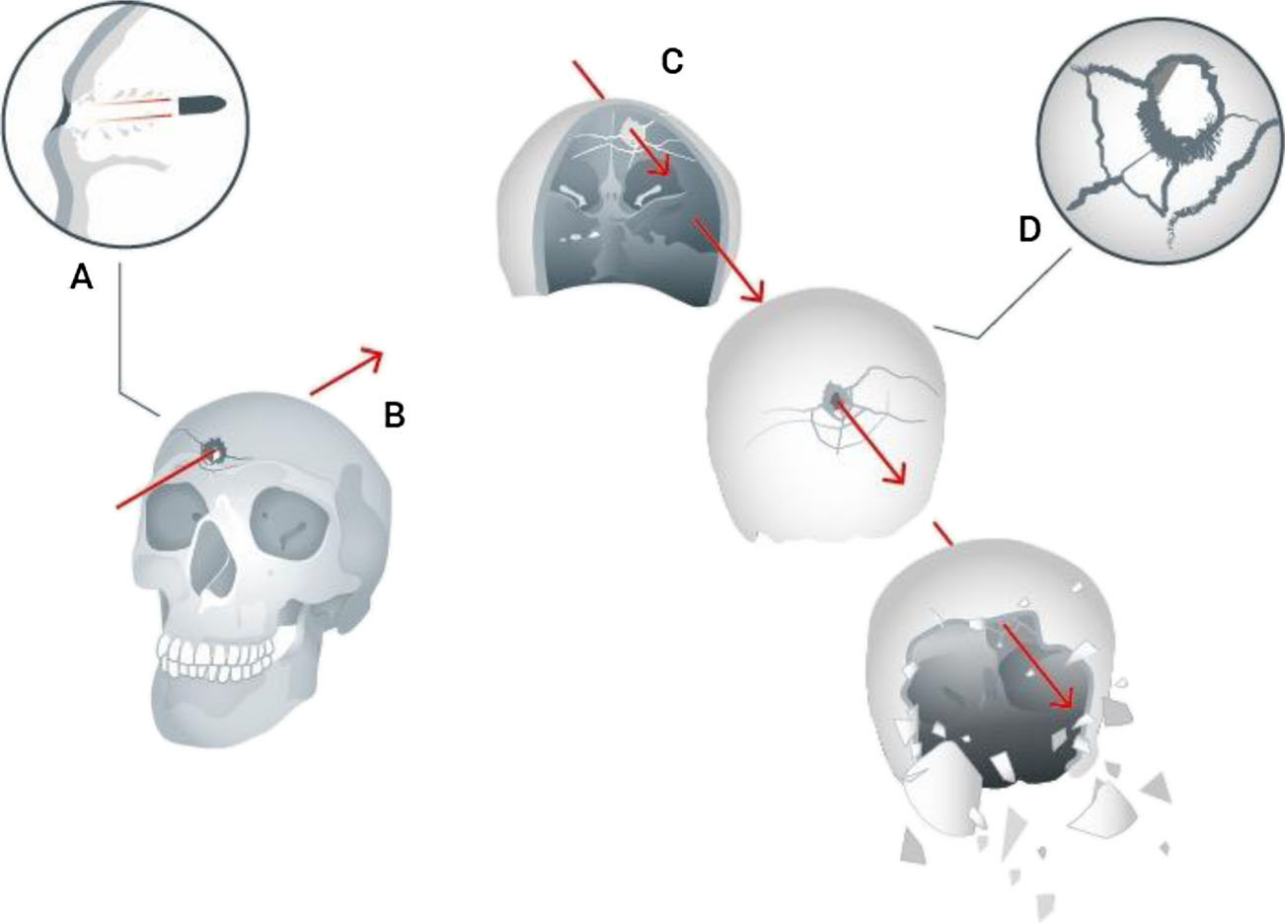
*a* Detail of entry wound (after DiMaio [18]). *b* Impact and passage of bullet through skull—front view (PFM). *c* Passage of bullet through skull—rear view—and development of secondary and tertiary fractures plus explosive comminution (PFM). *d* Detail of exit from cranial cavity (after Karger [22])

A bullet penetrating the skull typically creates a round to oval shaped hole in the outer table of the bone with a large bevelled out hole on the inner table. The outer table defect usually has sharp edges with a ‘punched out’ appearance, and the inner table defect has an ‘excavated’ cone like appearance (Fig. 6). If a bullet has sufficient energy to exit the cranial cavity, a similar process occurs, except that the inner table is now the ‘entrance’ surface and the outer table the ‘exit’. Atypical appearances do occur including bevelling of entrance wounds [21].

Smith et al. [10] reported that the flat SYNBONE® samples and empty SYNBONE® spheres shot with both modern and obsolete ammunition types produced bevelled defects with similarities to those seen in real flat bone but lacked the complexity produced in real crania and that this was unsurprising given the differences in structure between real bone and the polymers used in the artificial bones. The same effect is seen here where the polyurethane material used for the skulls does not reflect the complex structure of actual cranial bone.

The bullet strike may cause direct secondary radial fractures originating from the impact sites [22]. In addition, the rapid rise in intracranial pressure from the temporary cavity in the brain tissue can cause indirect tertiary concentric fractures. If the pressures are high enough, an ‘explosive’ injury will be produced with skull comminution [18, 22]. These features are summarised in Fig. 6. The secondary and tertiary fractures in high-energy strikes can cause fragmentation of the original penetrating defects making assessment of which was the entry and exit wound complicated.

### Limitations of the model

As described by Thali [7], a synthetic model that produces realistic injury patterns would be very useful for forensic reconstructions and be free of the ethical issues and biological variation inherent in using animals and cadaveric specimens [10].

The model used in the current work does have the disadvantage of the post mortem ‘cut’ line which is an inherent part of the manufacturing process (Email communication ARRK/Carr July 2016). As described by Viel et al. [23], applying *Puppe’s rule* in relation to ballistic skulls fractures, the pre-existing cut line impacts on fracture propagation within the model. Approaches to managing this are being explored.

Unlike Thali’s model [7], the one used in this work did not have a synthetic layer to simulate skin. A number of approaches to simulate skin have been reported in the literature [24], and work is ongoing to develop a suitable skin and soft tissue layers for this model.

### Caveats

This paper only reports findings with two variants of one ammunition type. Other weapon systems or ammunition types may produce different results under these experimental conditions.

## Conclusions

The aim of this work was to see if optimisation of an anatomically correct skull-brain model using simple simulants (polyurethane and gelatine or PermaGel™) would produce clinically realistic ballistic injury fracture patterns. At least one assessor out of five felt that the fracture pattern was close to real injury in over half of the models. Generally, the exit wounds were thought to be more realistic than the entry wounds. The model does have a number of limitations, and future work is planned to address the bonding between the two parts of the skull along with building realistic skin and tissue layers.

## Appendix.

**Table 7:** Skulls with more than one Likert-type score for fracture pattern of 3 or 4

Skull (Letter or number): Skull polymer: Skull fill: (Gelatine % or PermaGel™)	Score and comments assessor 1	Score and comments assessor 2	Score and comments assessor 3	Score and comments assessor 4	Score and comments assessor 5
C:UP5690:10	3: No comment.	2: Some entry radiating fractures. Exit wound bevelling, local comminution but insufficient fracture.	2: Entry—some radiating fractures. Exit—fracture pattern does not look like expected exit wound, i.e. multiple fragments ‘blown out’ with radial fractures from main part of damage but has been affected by cut/moulding line.	3: No comment	2: Entry—Not as much vault or base comminution in relation to entrance as would expect to see; suspect has been affected by entrance being just below saw cut. Some extension inferiorly into right orbit. Exit—Fragmentation at exit area quite representative but would expect extension inferiorly into base.
G:PU8098:10	3: No comment	3: Entry wound—radiating fracture lines; fracture to maxilla and base of skull. Exit wound—good radiating fractures but less comminution.	1: Entry—right frontal wound; skull seems to have fractured away through right orbit. Superior half of mould has cracked away. Exit—some fragment loss posteriorly.	3: If it had not been weakened by the artificial join (cut line), the fracture pattern around the entry and exit holes would be correct.	2: Entry—comminution of anterior fossa below entrance and extension into middle fossa with bone loss quite realistic but no extension across midline and no comminution of anterior vault (saw cut). Exit—vault comminution around exit but some of the fracture lines too regular. No extension into base posteriorly.
1:MU51:10	3: Difficult to reconstruct—perhaps comminution a bit extreme for range	3: Entrance—fracture lines with comminution. Exit—massive comminution, maybe too much.	1: Over 20 fragments. Bottom half of skull unaffected. Entry—to the frontal area but then top half of model has cracked into multiple parts. Exit—unable to comment on loss of ‘bone’ posteriorly.	1: does not feel right that the neurocranium fractured in isolation and so extensively	3: Vault path. Entry—entrance partly preserved. Exit—point not precisely seen. Saw cut prevents extension to skull base but otherwise realistic.
3:MU51:10	3: Good radiating lines around entrance; significant comminution of occiput.	3: Entry—multiple radiating fracture lines. Exit—bullet wipe marks* with comminution	2: Anterior entry with associated fracture in frontal bone. Exit—multiple fragments in occipital bone; would expect this to be held together by soft tissues which would clarify the exact fracture pattern.	2: entry fracture looks correct other than the artificial cut line; exit fracture pattern too large.	2: Entry—entrance incomplete superiorly and inferiorly with associated bone loss terminated inferiorly by saw cut. Some radiating fracture lines but pattern of bone loss unusual. Exit—exit much more realistic; multifragmentary but does not extend into skull vault due to saw cut. Would also expect some extension into middle fossa.
8:MU51:3	3: Reconstruction could move this up to a 4	4: Entry—fracturing out into orbit Exit—extensive comminution and distant fracture lines.	2: Left frontal entry wound. Fracture involves left orbit. Large fracture through left temporal bone—too many fragments. Exit—whole of posterior skull looks fragmented.	2: too broken	2: Entrance at 11:00—similar to others. No vault involvement and defect connected by fracture at posterior part ant fossa but no obvious radiating fractures. Exit—more realistic. Heavily fragmented; base and vault, precise point not identified.
11:MU51:10	3: Would look more convincing following reconstruction	4: Entry—eminating fracture lines; undisplaced frontal fractures. Exit—massive comminution with widespread fractures into base of skull/occiput.	2: Right frontal entry just on cut/mould line. Superior half of model generally intact. Significant injury but too many fragments. Exit—significant disruption, over 20 fragments. Complete destruction of posterior fossa.	2: too destroyed	2: Entrance—bordering on cut line anteriorly. No extension into vault. Ipsilateral involvement anterior fossa but no extension across midline. Exit—multifragmentary exit more realistic.
12:MU51:10	3: No comment	1: Entry—not seen a single fracture line from entry wound. Exit—too well marginated and minimal fracturing.	3: Right frontal entry—right orbital injury as expected producing orbital fracture. Exit—fragments blown out with associated fracture running parallel and then crossing sagittal suture. Fractures bounded by casting lines.	4: fracture pattern correct; only the entry hole looks too well circumscribed.	2: Entry—radiating fractures into base and vault, not quite as extensive Exit—radiating fractures into base and vault, not quite as extensive
21:MU51:PG	3: No comment	3: Entry—minimal fracture. Exit—external bevelling. Radiating fracture to foramen magnum and calvarium comminution.	3: PermaGel in place. Entry—small wound right frontal area. Exit—multiple fragments posteriorly equally above and below the casting/cut line. Fracture line on skull continuing from where fragments have fallen out.	2: entry fracture pattern looks too small; rear exit fractures look right.	2: Entry—external and Internal bevelling. Would expect more comminution Exit—extensive comminution and vault/base extensions more realistic but would expect more comminution and extension to foramen magnum: almost a ‘3’ for the exit.
23:MU51:PG	2: hovering between 1 and 2	1: Not the expected fracture pattern at entry or exit.	3: Entry—small fragment. Exit—small fragments blown out posteriorly, bounded by casting line.	3: size of entry point realistic but can see demarcation of cut line. Size of exit realistic.	1: Entry—superior defect but no inferior radiation to base. No vault extension. Exit—pattern of fragmentation not realistic and limited by saw cut. No radiation into distant vault.
25:MU51:PG	2: lack of radiation from entrance	3: Entry—internal bevelling, minimal fracture patterns. Exit—external bevelling; some radiating fractures and comminution. Needs more exit fractures!	3: Fractures have been altered by moulding/cut line. Entry—right frontal. Exit—small fragments posteriorly from small exit wound with stellate fracturing surrounding.	2: sizes look correct but there is no fracture propagation and false demarcation around cut line.	1: Entry—defect at 11:00 but shows no other radiating fractures. Exit—near limb of lambdoid; fracture does extend towards sagittal suture-? minimal diastasis. Not as much fragmentation as would expect, limited by saw cut.
26:MU51:7	3: radiation from entrance stopped by cut line	3: Entry—some emanating fracture and distant (right orbit/maxilla) fracture but no comminution. Exit—comminution and obliquity of fracture margins in keeping.	3: Some fracture lines across temporal bones. Entry—anterior entry point with associated radial fractures into frontal bone and orbit. Exit—widespread posterior injury with multiple fragments ‘blown out’	4: Commenting primarily on the entry point this is the closest I have seen in terms of entry hole size and fracture pattern.	2: Entry—bevelling quite prominent. Cleaved obliquely but not crossing midline, stopped by saw cut so no vault involvement. Defect at post ant fossa away from entrance but comminuted by fracture. Similar in appearance to other cases seen here but not a typical appearance in real life. Exit—more realistic. Multifragmenting.
27:MU51:7	2: perhaps with reconstruction closer to a 3	3: Entry—local comminution, reverse bevelling, some fractures into face. Exit—external bevelling with comminution.	1: Appears to be too many fragments to be realistic—shattered appearance. Entry—small right frontal entry wound with loss of right frontal bone. Exit—multiple smaller fragments involving posterior fossa.	3: No frontal sinus (in the model) so no fracture	3: Entry—entrance at saw cut but does produce base and vault involvement. Similar post anterior fossa defect as with others. Exit—heavily fragmented exit with: vault and base. Precise point not seen.
28:MU51:7	3: No comment	3: Entry—some comminution, limited radial fractures. Exit—bevelling present, comminution with extending fracture lines.	3: Entry—frontal wound in area of frontal air sinuses. Some bone fracturing. Exit—posteriorly widespread loss of bone. Possible stellate pattern of fracture. Fracture extends down to foramen magnum.	2: Entry and exit points incorrect due to cut line	2: Entry—some fragmentation superiorly but not passing through saw cut. No radiation into skull base. Exit—area more realistic. Multifragmenting. Base involvement including radiation to foramen magnum. Precise point not identified.

Comments summarised where available

* Bullet wipe is the debris left by the bullet ( lubricant, propellant and metal traces) around the entrance site.
